# The adrenal steroid profile in adolescent depression: a valuable bio-readout?

**DOI:** 10.1038/s41398-022-01966-2

**Published:** 2022-06-18

**Authors:** Raphael Hirtz, Lars Libuda, Anke Hinney, Manuel Föcker, Judith Bühlmeier, Paul-Martin Holterhus, Alexandra Kulle, Cordula Kiewert, Berthold P. Hauffa, Johannes Hebebrand, Corinna Grasemann

**Affiliations:** 1 Division of Pediatric Endocrinology and Diabetology, Department of Pediatrics II, University Hospital Essen, University of Duisburg-Essen, Hufelandstr. 55, 40211 Essen, Germany; 2 Department of Child and Adolescent Psychiatry and Psychotherapy, University Hospital Essen, University of Duisburg-Essen, Wickenburgstr. 21, 40211 Essen, Germany; 3 Institute of Nutrition, Consumption and Health, Faculty of Natural Sciences, University Paderborn, Warbuger Str. 100, 33098 Paderborn, Germany; 4 Department of Child and Adolescent Psychiatry, University Hospital Münster, Schmeddingstr. 50, 48149 Münster, Germany; 5 Department of Paediatrics I, Paediatric Endocrinology and Diabetes, University Hospital of Schleswig-Holstein, UKSH, Campus Kiel, and Christian-Albrechts-University, Arnold-Heller-Str. 3, 24105 Kiel, Germany; 6 Department of Pediatrics, Division of Rare Diseases, St Josef-Hospital, and CeSER, Ruhr-University Bochum, Alexandrinenstr. 5, 44791 Bochum, Germany

**Keywords:** Physiology, Diagnostic markers, Depression

## Abstract

There is preliminary evidence that adrenal steroids other than cortisol may be valuable biomarkers for major depressive disorder (MDD). So far, studies have been conducted in adults only, and conclusions are limited, mainly due to small sample sizes. Therefore, the present study assessed whether adrenal steroids serve as biomarkers for adolescent MDD. In 261 depressed adolescents (170 females) treated at a single psychiatric hospital, serum adrenal steroids (progesterone, 17-hydroxyprogesterone, 21-deoxycortisol, 11-deoxycortisol, cortisol, cortisone, deoxycorticosterone, corticosterone) were determined by liquid chromatography-tandem mass spectrometry. Findings were compared to that of an age- and sex-matched reference cohort (*N* = 255) by nonparametric analysis of variance. Nonparametric receiver operating characteristics (ROC) analyses were conducted to evaluate the diagnostic performance of single steroids and steroid ratios to classify depression status. Sensitivity analyses considered important confounders of adrenal functioning, and ROC results were verified by cross-validation. Compared to the reference cohort, levels of deoxycorticosterone and 21-deoxycortisol were decreased (*P* < 0.001). All other glucocorticoid- and mineralocorticoid-related steroids were increased (*P* < 0.001). The corticosterone to deoxycorticosterone ratio evidenced excellent classification characteristics, especially in females (AUC: 0.957; sensitivity: 0.902; specificity: 0.891). The adrenal steroid metabolome qualifies as a bio-readout reflecting adolescent MDD by a distinct steroid pattern that indicates dysfunction of the hypothalamus–pituitary–adrenal axis. Moreover, the corticosterone to deoxycorticosterone ratio may prospectively qualify to contribute to precision medicine in psychiatry by identifying those patients who might benefit from antiglucocorticoid treatment or those at risk for recurrence when adrenal dysfunction has not resolved.

## Introduction

Depression is a debilitating and highly prevalent psychiatric disorder affecting as many as 20% of teenagers at some point during adolescence [1]. Thus, there is considerable interest in biomarkers of major depressive disorder (MDD) to identify individuals at risk for MDD or its recurrence and provide target therapy by deliberate classification [2]. In this regard, much effort has been devoted to studying the relationship between cortisol and MDD, specifically to identify subtypes of depression [3] and to predict the risk for recurrence [4]. In a meta-analysis of 48 studies, Juruena, Bocharova [3] identified differences in cortisol levels and the responsiveness of the hypothalamus–pituitary–adrenal (HPA) axis to pharmacological challenges between patients with melancholic and atypical depression. However, meta-analyses also revealed that cortisol levels neither qualify to predict the treatment response to psychological therapy nor to antidepressants [5].

**Fig. 1:**
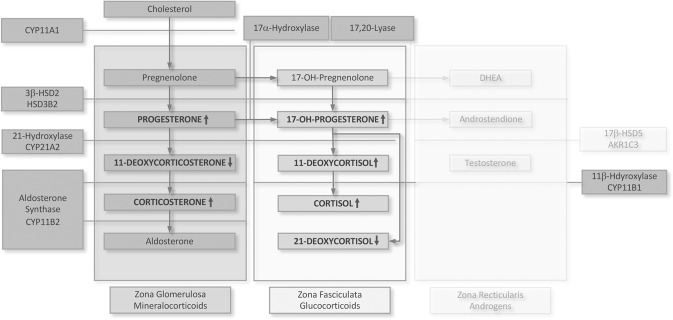
Pathways of steroid hormone synthesis in the adrenal glands, including the involved enzymes and the genes coding these enzymes (boxes surrounding the three adrenal zones) adapted from Han, Walker [52]. Steroid hormone levels altered in adolescent MDD compared to the reference cohort are printed in all capitals and bold type. As androgens were not studied, their synthesis is grayed out. CYP cytochrome P450, HSD hydroxysteroid dehydrogenase, AKR1C3 aldo-keto reductase family 1 member C3.

However, preliminary evidence suggests that adrenal steroids other than cortisol may contribute to precision medicine in psychiatryic disorders. For example, two studies reported hypersecretion of 11-deoxycortisol, a precursor in the synthesis of cortisol (see Fig. 1), in adult depression [6, 7]. Moreover, a series of studies relying on stimulation and suppression tests identified increased corticosterone secretion in MDD [6, 8], which has been suggested to be an additional biochemical criterion in the diagnosis of MDD by pharmacological challenges as the dexamethasone suppression test (DST) [6]. However, these studies comprised only a limited number of patients ranging from 6 to 36 and mainly relied on measurements of a (very) limited number of adrenal steroids. Moreover, in some of these studies, steroid measurements were solely based on radioimmunoassays, which are susceptible to cross-reactivity with other steroids [9]. Thus, although promising, these findings need confirmation.

In addition to the potential of the adrenal steroid metabolome as a diagnostic tool in MDD, it may also help to provide insights into the pathophysiology of MDD. Tetrahydrodeoxycorticosterone (THDOC), a neuroactive steroids that primarily modulates central nervous system (CNS) activity by rapid, mostly non-genomic actions [10], has been related to depression [11]. While some studies have shown increased THDOC levels in MDD [12, 13], its adrenal precursor deoxycorticosterone has hardly been studied in mood disorders apart from postpartum depression [14]. Considering that increased THDOC levels may indicate an impaired inhibitory HPA axis feedback loop [15], this information may also be studied at the adrenal level by analyzing its precursor deoxycorticosterone.

Also, the combined examination of multiple steroids may provide additional information regarding clinical implications not obtainable by single steroid analysis. For example, Goodyer et al. showed that an increased cortisol to DHEA ratio is associated with a prospective diagnosis of MDD as well as its persistence in adolescents [16]. However, research examining other steroid ratios in this regard has not been conducted.

This cross-sectional study was intended to assess the utility of multiple individual serum steroids (progesterone, 17-hydroxyprogesterone, 21-deoxycortisol, 11-deoxycortisol, cortisol, cortisone, deoxycorticosterone, corticosterone) and selected steroid ratios indicative of adrenal enzyme activity as a bio-readout of MDD by comparing a large clinical sample of depressed adolescents with age- and sex-matched reference cohort. Steroid analysis in this study was performed based on the reference method for steroid hormone analysis, that is liquid chromatography-tandem mass spectrometry (LC-MS/MS). Considering the above review of previous studies, we hypothesized adrenal dysfunction affecting glucocorticoid- and mineralocorticoid-related steroids in adolescent MDD.

## Methods

### Participants—psychiatric sample

Data of psychiatric patients were derived from the baseline assessments of a two-armed parallel-group, double-blind RCT, which investigated the effect of 25-hydroxyvitamin D3 deficiency (25(OH)D3 ≤30 nmol/l [equivalent to ≤12 ng/ml]; DRKS00009758) on depressive symptoms in inpatients or daycare patients treated at the Department of Child and Adolescent Psychiatry, Psychosomatics and Psychotherapy Essen (LVR-Klinikum Essen), Germany. In addition, data from a cross-sectional study focusing on the relationship between nutrition and mental disorders (“Nutrition and Mental Health Study”) were used. Considering that both studies followed the same protocol, data were pooled. The studies were conducted in accordance with the Declaration of Helsinki and approved by the local Ethics Committee (No. 15-6363-BO). Informed written consent was obtained from patients as well as their (foster) parents when patients were below 18 years of age. Psychiatric patients were eligible for inclusion if aged 11–18.9 years. Exclusion criteria were a concurrent diagnosis of severe somatic disease and/or intellectual disability [17].

### Participants—reference cohort

Primary data (*N* = 573) from the norming sample of the LC-MS/MS assay for the analysis of C21 steroids (progesterone, 17-hydroxyprogesterone, 21-deoxycortisol, 11-deoxycortisol, cortisol, cortisone, deoxycorticosterone, corticosterone) from Kulle, Welzel [18] was used to set up the reference cohort [18]. In short, the norming sample was based on leftover blood draws before minor surgery or from diagnostic blood draws in pediatric subjects with no evidence of active endocrine or systemic disease upon comprehensive evaluation. Moreover, subjects were free of steroid medication and in the follicular phase of the menstrual cycle, if applicable [18]. Other information related to the reference cohort’s demographic, anthropometric, or psychosocial characteristics was not available.

### Questionnaires

In psychiatric patients, the Beck Depression Inventory-II (BDI-II) was used to assess the severity of depressive symptoms. The BDI-II is a self-reported questionnaire that records depressive symptoms according to DSM-IV diagnostic criteria for major depressive disorder (MDD) over the past two weeks by 21 items [19]. Answers are scored on a 4-point Likert scale (0-3), with higher scores indicating a greater degree of depression [19]. Total scores between 14–19 indicate mild, between 20–28 moderate, and above 28 severe depressive symptoms [19]. Patients with a total BDI-II score above 13 were classified as depressed.

Psychiatric diagnoses were established either via the semi-structured interview ‘Schedule for Affective Disorders and Schizophrenia for School-Aged Children—Present and Lifetime Version’ (K-SADS-PL) according to DSM-IV (94.1% of patients) or via clinical assessment according to ICD-10 (5.9%) when no K-SADS-PL was performed.

In addition, covariates known to affect adrenal steroid levels, including the intake of psychotropic medication, health-related behavior such as smoking, and the use of COC, were recorded on admission. Moreover, the socioeconomic status (SES) was captured by the household net income, parental education, and occupation [20], also as a measure of adverse life events [21].

### Anthropometric measures

Psychiatric patients were subjected to a physical examination upon admission, including an assessment of body height and body weight. Height was determined in upright posture to the nearest 0.1 cm using a wall-mounted stadiometer. Body weight was measured in underwear with an electronic scale to the nearest 0.1 kg. BMI was determined by the ratio of weight in kg and the height in meters squared (kg/m^2^). To consider the effect of age, BMI was z-transformed according to percentile charts for German children and adolescents (RefCurv Version 0.4.4, https://refcurv.com [22]).

### Laboratory studies

In the reference cohort, blood was drawn in the morning before 10 am (for further details, please refer to Kulle, Welzel [18]) and immediately stored at −20 °C until steroid hormone analysis from plasma samples by LC-MS/MS [18]. In psychiatric patients, venipuncture was performed within the first 3 days after admission in the morning before 10 am after an overnight fast. After sampling, blood was transferred within one hour to the laboratory of the University Hospital Essen for analyses, and serum aliquots were stored at −80 °C until LC-MS/MS analysis. Importantly, steroid hormones are stable for a long time when stored at low temperatures [23].

In brief, for LC-MS/MS analysis, the internal standard mixture was combined with the stored sample as well as the calibrator and control aliquots to monitor recovery. Samples were extracted using Oasis MAX SPE system Plates (Waters, Milford, MA, USA). LC-MS/MS was performed using a Waters Quattro Premier/Xe triple-quadrupole mass spectrometer connected to a Waters Acquity (Waters, Milford, MA, USA; Table 1 for details on assays). All analyses are accredited according to DIN EN ISO/IEC 15189 since 2013. Quality control procedures were the same for samples from the reference cohort and the psychiatric patients.

**Table 1:** Assays and their performance characteristics, including the abbreviations and systematic names of the steroids analyzed.

Steroid	Abbreviation	Systematic name (IUPAC)	Assay system	Assay type	Intra-assay variation	Total assay variation	Detection range
25(OH)-vitamin D	25(OH)D3	(3*S*,5*Z*,7*E*)−9,10-secocholesta-5,7,10-triene-3,25-diol	Siemens ADVIA Centaur^a^	CLIA	<5.3%	<11.9%	10.5–375 nmol/L
Estradiol	E2	(17β)-estra-1,3,5(10)-triene-3,17-diol	Siemens ADVIA Centaur	CLIA	<11.2%	<13.3%	43.6–11,010 pmol/l
Progesterone	P	pregn-4-ene-3,20-dione	Waters Acquity UPLC System^b^	LC-MS/MS	<6.3%	<8.5%	0.1–200 nmol/L
17-hydroxyprogesterone	17OHP	17-hydroxypregn-4-ene-3,20-dione	Waters Acquity UPLC System	LC-MS/MS	<4.3%	<8.5%	0.1–200 nmol/L
Deoxycorticosterone	DOC	21-hydroxypregn-4-ene-3,20-dione	Waters Acquity UPLC System	LC-MS/MS	<6.6%	<7.8%	0.1–200 nmol/L
Corticosterone	B	11β,21-dihydroxypregn-4-ene-3,20-dione	Waters Acquity UPLC System	LC-MS/MS	<4.0%	<8.6%	0.1–200 nmol/L
21-deoxycortisol	21S	11β,17-dihydroxypregn-4-ene-3,20-dione	Waters Acquity UPLC System	LC-MS/MS	<4.9%	<8.7%	0.1–200 nmol/L
11-deoxycortisol	11S	17,21-dihydroxypregn-4-ene-3,20-dione	Waters Acquity UPLC System	LC-MS/MS	<5.8%	<8.7%	0.1–200 nmol/L
Cortisol	F	11β,17,21-trihydroxypregn-4-ene-3,20-dione	Waters Acquity UPLC System	LC-MS/MS	<5.6%	<9.7%	1–2000 nmol/L
Cortisone	E	17,21-dihydroxypregn-4-ene-3,11,20-trione	Waters Acquity UPLC System	LC-MS/MS	<5.7%	<8.6%	0.1–200 nmol/L

*IUPA* international union of pure and applied chemistry, *CLI* chemiluminescent immunoassay, *LC-MS/M* liquid chromatography-tandem mass spectrometry.

^a^Siemens Healthineers, Erlangen, Germany.

^b^Waters, Milford, MA, USA.

### Statistical analysis

Data handling and analyses were conducted with SPSS 27.0 (Armonk, NY: IBM Corp.) or R (R core team, 2020) and the “Rfit” package [24]. All tests were performed controlling the two-tailed false-discovery rate (FDR) at *q* < 0.05, except for sensitivity analyses detailed below.

For statistical analysis, all depressed (BDI-II score >13) male psychiatric patients with complete information on all variables of interest were considered for analysis. However, for reasons of comparability with the reference cohort as well as steroid hormone levels in males, only those depressed female patients in the follicular phase of the menstrual cycle (estradiol <734 pmol/l, progesterone <6.36 nmol/l [25]) and complete information were included. Moreover, female patients taking combined oral contraceptives (COCs), as well as all patients with a diagnosis of anorexia nervosa, were excluded from the present analysis due to potential effects on steroid metabolism [26, 27].

Subjects from the reference cohort were selected according to the distribution of age and sex in the sample of psychiatric patients to consider the effects of both variables on adrenal steroid levels. For this purpose, the largest possible random subsample of the reference cohort was chosen using the “Complex Samples” procedure as part of SPSS.

### Robust analysis of variance

**Fig. 2:**
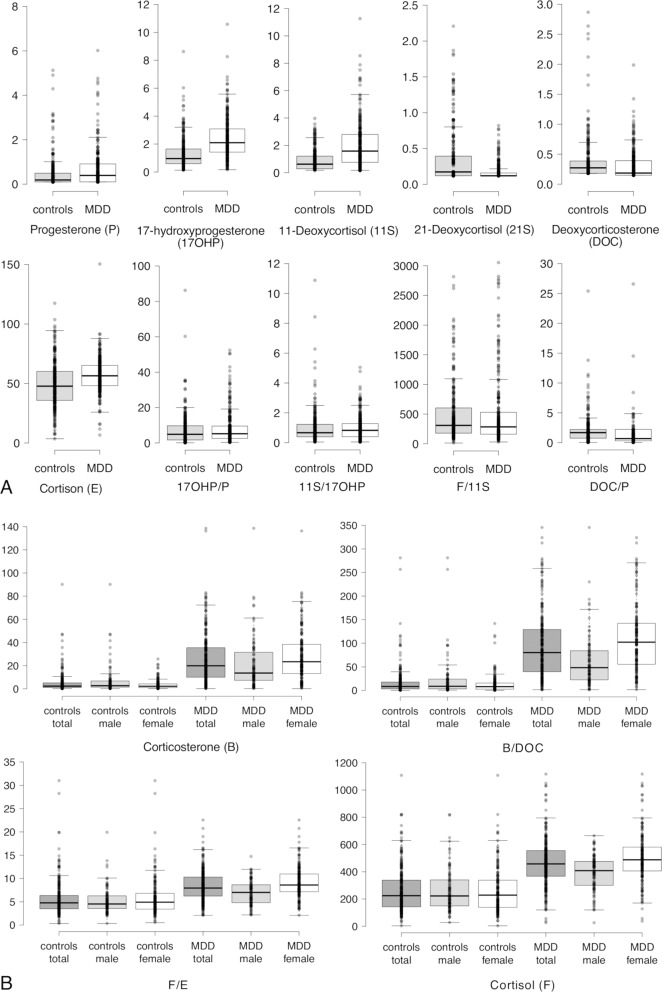
Boxplots for all steroids and steroid ratios. Panel (**A**) displays steroids and steroid ratios with, panel (**B**) without a significant interaction with sex, separately for the reference cohort and adolescents with MDD (*x* axis). The *y* axis corresponds to nmol/l concerning single steroids and is unit-free concerning steroid ratios. Note, extreme outliers (exceeding three times the interquartile range) are not displayed for values of B and the ratios of cortisol/11-deoxycortisol, deoxycorticosterone/progesterone, and corticosterone/deoxycorticosterone to avoid a distortion of the *y* axis and the graphical representation in the lower range of values. The full range of values, including extreme outliers, is shown in Supplementary Fig 1.

Single steroids but also steroid ratios of interest (Table 3, dependent variables [DV]) were found to be non-normally distributed with a variable number of outliers as identified by Shapiro–Wilk tests, visual inspection of Q–Q plots, and boxplots (Fig. 2 and Supplementary Fig 1). Considering that common data transformations did not alleviate non-normality, and in the presence of persisting outliers, a nonparametric, rank-based analysis of variance [24, 28] (ANOVA) robust to outliers and insensitive to violations of normality was performed to compare the reference sample with the sample of psychiatric patients (independent variable 1 [IV_1_]: sample). To consider a potentially moderating effect of sex (IV_2_) on steroid levels comparing both samples, an additional two-way interaction term (IV_1_ x IV_2_) was included in the statistical model.

Note, currently, no option is implemented to calculate effect sizes for this ANOVA approach but considering either a conventional parametric or nonparametric comparison of central tendency, power (1-β) would have been sufficient (≥0.8) for an effect size as small as *d* = 0.25 (assuming α = 0.05) in either instance (GPower 3.1).

### Receiver operating characteristics (ROC) analysis

Nonparametric ROC analyses were conducted to evaluate the performance of single steroids but also steroid ratios to classify group membership (psychiatric vs. reference cohort), separately for sex if there was a significant interaction between sample and sex revealed by the preceding step of analysis of variance.

As a performance measure, the area under the curve (AUC) was determined and either tested against a random classifier at chance level (AUC = 0.5) or compared between sexes when an interaction as outlined above was present. Testing relied on a test statistic with approximate normal distribution that results from the asymptotic properties of the AUC. The AUC was converted to Cohen’s *d* according to Ruscio [29] for reasons of familiarity with the latter and ease of effect size interpretation (*d*: small ≥0.2, medium ≥0.5, large ≥0.8).

The optimal cut-off point was chosen according to the maximum Kolmogorov–Smirnov statistic, the largest difference between sensitivity (true positive rate) and 1-specificity (false positive rate).

### Sensitivity analysis

Considering that information on potential confounders (smoking, psychotropic medication, BMI, and SES) were only available for the psychiatric sample, their impact on adrenal steroids and their ratios was examined by Kendall’s τ_b_, a rank-based correlation with reasonable robustness against outliers [30] (effect size *r*: small ≥0.1, medium ≥0.3, large ≥0.5 [29]). In addition, 25(OH)D3 levels were included for confounder analysis, as 42.5% of psychiatric patients were 25(OH)D3 deficient.

Moreover, to explicitly consider the effects of potential confounders on the comparison between steroid hormone levels, a subsample of psychiatric patients was defined by excluding those patients that reported smoking and the use of psychotropic medication on admission (subsequently “restricted psychiatric sample”). Also, this subsample only included patients with a BDI-II score above 13 and a diagnosis of MDD verified by the K-SADS-PL or clinical assessment when no K-SADS-PL was performed. For a comparison with the restricted psychiatric sample, eligible subjects from the reference cohort were selected as outlined above for the total psychiatric sample.

ROC results were confirmed by simple two-fold cross-validation and a split between the test and verification sample of 70% and 30%, respectively. For cross-validation, the AUC between the test and verification sample was compared as outlined above by a test statistic with approximate normal distribution.

The potential effect of hospitalization on steroid hormone levels was investigated by comparing cortisol, used as a marker steroid for hospitalization-related stress [31], between inpatients and daycare patients by the rank-based approach outlined above.

Note, all sensitivity analyses were considered exploratory as they were only conducted to verify the main findings of the present study and, therefore, not corrected for multiple comparisons.

## Results

### Descriptives

Altogether, 261 psychiatric patients and 255 subjects from the reference cohort were included for analysis. The mean age of psychiatric patients was 15.7 (SD 1.6) years and the mean BDI-II score 29.0 (SD 9.9), indicating severe depressive symptoms. The psychiatric sample included almost twice as many girls (65.1%) as boys. On admission, 22.6% of patients were smokers and 22.2% were taking psychotropic medication, including antidepressants (Table 2).

**Table 2:** Patient characteristics.

	Psychiatric sample (*N* = 261)	Psychiatric sample restricted (*N* = 159)	Controls^a^ (*N* = 255)
Age	15.72 (1.57) [11.80–18.83]	15.49 (1.58) [11.80–18.83]	#
Age category (%)			
7–≤13 years	5.7	6.3	5.9
13–≤16	46.0	52.2	45.9
16–18	48.3	41.5	48.2
Gender (female %)	65.1	69.2	65.1
z-BMI	0.03 (1.48) [−4.93–2.76]	−0.04 (1.58) [−4.93–2.76]	#
BDI-II	28.95 (9.90) [14–59]	27.7 (9.28) [14–54]	#
BDI-II severity category (%)			
Mild (sum score 14–19)	18.8	22.0	#
Moderate (20–28)	35.6	36.5	#
Severe (>28)	45.6	41.5	#
Psychotropic medication (%)	22.2	#	#
Smoking (%)	22.6	#	#
25(OH)D3	37.43 (18.03) [11.23–106.58]	38.72 (19.51) [11.23–106.58]	#
25(OH)D3 < 30 nmol/l (%)	42.5	42.2	#

Provided are the mean, standard deviation (in round brackets), and range (in square brackets) for interval scaled variables and percentages otherwise; *z* z-standardized. *#* no information available/does not apply.

^a^Compiled according to the distribution of age and sex of the psychiatric sample without restrictions.

### ANOVA

There was a significant mean difference in C21 steroids and their ratios between the psychiatric and reference cohort, except for the ratios of 17-hydroxyprogesterone/progesterone (*P* = 0.39), 11-deoxycortisol/17-hydroxyprogesterone (*P* = 0.53), and cortisol/11-deoxycortisol (*P* = 0.37) (Tables 3 and 4, Figs. 1 and 2, and Supplementary Fig 1). Levels of deoxycorticosterone (*P* < 0.001) and 21-deoxycortisol (*P* < 0.001), and the ratio of deoxycorticosterone/progesterone (*P* < 0.001) were significantly decreased in psychiatric patients compared to subjects from the reference cohort.

**Table 3:** Steroid and steroid ratios—results.

	Psychiatric sample (*N* = 261)		Psychiatric sample restricted (*N* = 159)		Controls^a^ (*N* = 255)	
	Mean_robust_ (SD) [range]	Median (IQR)	Mean_robust_ (SD) [range]	Median (IQR)	Mean_robust_ (SD) [range]	Median (IQR)
Progesterone (P)	0.44 (0.90) [0.10–6.02]	0.40 (0.10–0.92)	0.41 (0.92) [0.10–6.02]	0.36 (0.10–0.91)	0.22 (0.74) [0.10–5.13]	0.19 (0.10–0.50)
17-hydroxyprogesterone (17OHP)	2.19 (1.49) [0.17–10.58]	2.10 (1.41–3.10)	2.12 (1.44) [0.23–10.58]	2.07 (1.35–2.93)	1.05 1.14) [0.13–8.63]	0.96 (0.59–1.65)
Deoxycorticosterone (DOC)	0.19 (0.25) [0.15–1.99]	0.19 (0.15–0.39)	0.19 (0.27) [0.15–1.99]	0.18 (0.15–0.37)	0.27 (0.39) [0.18–0.29]	0.27 (0.18–0.39)
Corticosterone (B)	21.53 (21.98) [0.24–138.65]	19.90 (9.94–35.53)	21.84 (22.98) [0.33–138.65]	19.63 (11.20–37.76)	2.65 (8.68) [0.21–90.20]	2.34 (1.18–5.21)
21-deoxycortisol (21 S)	0.17 (0.12) [0.12–2.21]	0.12 (0.12–0.16)	0.17 (0.13)^#^ [0.12–0.82]	0.13 (0.12–0.13)	0.18 (0.40) [0.12–2.21]	0.17 (0.12–0.39)
11-deoxycortisol (11 S)	1.70 (1.67) [0.17–11.26]	1.58 (0.75–2.80)	1.63 (1.72) [0.17–11.26]	1.56 (0.73–2.75)	0.69 (0.79) [0.17–3.97]	0.62 (0.28–1.21)
Cortisol (F)	457.68 (175.72) [26.11–1117.22]	457.51 (366.45–556.01)	468.02 (182.60) [26.11–1117.22]	470.27 (375.62–561.30)	229.78 (165.64) [3.22–1107.86]	224.38 (142.09–338.91)
Cortisone (E)	56.65 (14.45) [6.68–150.42]	56.48 (48.04–65.37)	56.42 (15.27) [6.68–150.42]	56.26 (47.39–65.56)	48.10 (19.40) [3.52–117.45]	47.76 (35.51–60.59)
17OHP/P	5.83 (9.43) [0.31–52.44]	5.21 (2.58–9.62)	5.70 (10.14) [0.31–52.44]	5.07 (2.48–9.84)	5.17 (9.17) [0.08–86.26]	4.88 (1.64–9.75)
DOC/P	0.83 (1.33) [0.03–7.67]	0.69 (0.31–1.50)	0.87 (11.61) [0.04–7.55]	0.72 (0.30–2.34)	1.49 (2.39) [0.04–24.24]	1.68 (0.75–2.16)
B/DOC	82.03 (90.81) [0.31–281.05]	80.32 (38.74–129.27)	88.51 (98.26) [0.31–281.05]	87.33 (41.38–136.96)	9.24 (31.07) [0.31–281.05]	8.35 (3.18–17.80)
11S/17OHP	0.82 (0.82) [0.05–5.05]	0.83 (0.40–1.29)	0.85 (0.90) [0.05–5.05]	0.84 (0.40–1.40)	0.75 (1.17) [0.04–10.89]	0.67 (0.39–1.24)
F/11S	296.98 (763.15) [30.22–6175.68]	284.09 (160.20–533.23)	310.67 (815.70) [30.22–6175.68]	286.66 (172.58–596.09)	327.60 (576.21) [12.39–4259.38]	308.98 (177.90–607.13)
F/E	8.14 (3.31) [2.09–22.56]	7.95 (6.22–10.31)	8.43 (3.39) [2.18–22.56]	8.38 (6.35–10.90)	4.86 (3.74) [0.34–31.01]	4.77 (3.48–6.41)

Robust mean according to Huber, standard deviation (round brackets), and range (square brackets) as well as the median and the interquartile range (IQR: 25th to 75th percentile; in round brackets) for single steroids and steroid ratios of interest. *#* “standard” mean, no robust mean available.

^a^Results for the control sample chosen according to the distribution of age and sex in the total psychiatric sample.

**Table 4:** Ranked ANOVA results.

	Sample		Sex		Sample x Sex	
Steroid	*F*	*P* value	*F*	*P* value	*F*	*P* value
Progesterone (P)	16.12	<0.001	10.32	0.001	0.03	0.85
17-hydroxyprogesterone (17OHP)	123.68	<0.001	64.24	<0.001	3.75	0.05
Deoxycorticosterone (DOC)	25.39	<0.001	7.41	0.007	0.50	0.48
Corticosterone (B)	386.54	<0.001	25.62	<0.001	44.72	<0.001
21-deoxycortisol (21 S)	24.07	<0.001	2.92	0.09	2.92	0.09
11-deoxycortisol (11 S)	83.71	<0.001	4.49	0.03	0.26	0.61
Cortisol (F)	195.10	<0.001	10.72	0.001	14.73	<0.001
Cortisone (E)	29.67	<0.001	4.00	0.046	0.62	0.43
17OHP/P	0.75	0.39	89.80	<0.001	2.68	0.10
DOC/P	17.31	<0.001	29.30	<0.001	2.57	0.11
B/DOC	447.62	<0.001	73.90	<0.001	96.33	<0.001
11S/17OHP	0.40	0.53	10.32	0.001	1.74	0.19
F/11S	0.82	0.37	10.39	0.001	0.03	0.87
F/E	118.63	<0.001	22.22	<0.001	12.58	<0.001

Results for the comparison of steroids and their ratios between psychiatric patients and controls (IV: sample); sample x sex = interaction between both IVs. Between group degrees of freedom (df_between_) = 1, within-group df 512 for all analysis except 21 S (df_within_ = 510).

An interaction between sample (psychiatric vs. reference cohort, IV_1_) and sex (IV_2_) was found for cortisol (*P* < 0.001; please see Supplementary Table 1 for detailed results), corticosterone (*P* < 0.001), and the ratio of corticosterone/deoxycorticosterone (*P* < 0.001) as well as cortisol/cortisone (*P* < 0.001), which was driven by depressed females that evidenced higher steroid hormone levels and ratios than depressed boys.

### ROC analysis

**Fig. 3:**
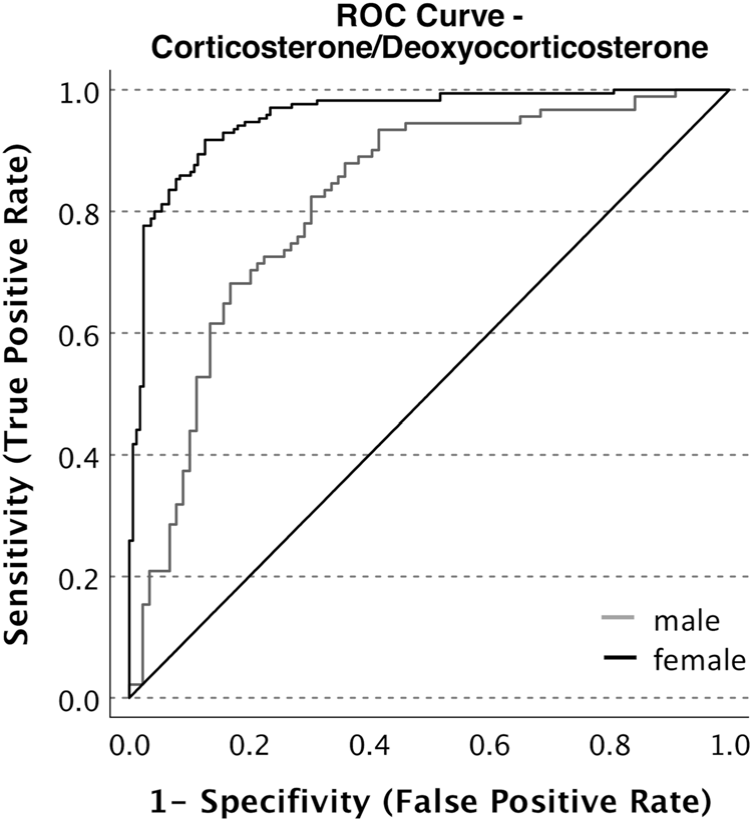
ROC curves for corticosterone/deoxycorticosterone. Results are separately displayed for males and females.

In line with results from the ANOVA analyses, all steroids and steroid ratios except for the ratios of 17-hydroxyprogesterone/progesterone (*P* = 0.09), cortisol/11-deoxycortisol (*P* = 0.50), and 11-deoxycortisol/17-hydroxyprogesterone (*P* = 0.90) outperformed a classification at chance level (Table 5). For 17-hydroxyprogesterone (AUC = 0.768), cortisol (AUC = 0.822), corticosterone (AUC = 0.893), and the ratios of cortisol/cortisone (AUC = 0.765) and corticosterone/deoxycorticosterone (AUC = 0.914) there was a large effect size, up to *d* = 1.93 for the corticosterone/deoxycorticosterone ratio with the best classification performance among all steroids and steroid ratios (Fig. 3). Concerning those steroids and steroid ratios with a significant interaction between sample and sex, there was a larger AUC for females than males for corticosterone (*P* < 0.001) and the corticosterone/deoxycorticosterone ratio (*P* < 0.001). Regarding neither steroid or steroid ratio, there was a significant difference between the AUC of the test and verification sample (Table 5), also in the restricted sample (Supplementary Table 2).

**Table 5:** ROC results.

	AUC (95%–CI)	*P* value	Cohen’s d	K–S statistic	Cut-off	Sensitivity	Specificity	AUC_CV_ (95%–CI)	*P* value_CV_
B_DOC									
Total	0.914 (0.884–0.944)	<0.001	1.93	0.696	32.44	0.801	0.894	0.900 (0.850–0.951)	0.645
Male	0.818 (0.741–0.896)	<0.001	1.28	0.539	10.04	0.949	0.59	0.804 (0.688–0.919)	0.836
Female	0.957 (0.934–0.981)	<0.001	2.43	0.792	27.55	0.902	0.891	0.947 (0.899–0.995)	0.699
Corticosterone (B)									
Total	0.893 (0.859–0.926)	<0.001	1.76	0.657	8.11	0.796	0.861	0.870 (0.811–0.930)	0.521
Male	0.790 (0.708–0.873)	<0.001	1.14	0.550	6.70	0.780	0.770	0.781 (0.660–0.902)	0.901
Female	0.940 (0.913–0.968)	<0.001	2.2	0.745	11.87	0.787	0.958	0.926 (0.867–0.985)	0.666
Cortisol (F)									
Total	0.822 (0.777–0.867)	<0.001	1.31	0.573	351.08	0.790	0.793	0.810 (0.740–0.879)	0.776
Male	0.748 (0.658–0,838)	<0.001	0.95	0.488	263.59	0.881	0.607	0.771 (0.648–0.895)	0.766
Female	0.853 (0.803–0.904)	<0.001	1.48	0.659	356.76	0.852	0.807	0.934 (0.751–0.917)	0.700
F/E									
Total	0.765 (0.715–0.816)	<0.001	1.02	0.502	6.98	0.813	0.827	0.768 (0.691–0.844)	0.959
Male	0.699 (0.606–0.793)	<0.001	0.74	0.351	5.55	0.695	0.656	0.717 (0.582–0.851)	0.838
Female	0.794 (0.734–0.854)	<0.001	1.16	0.592	6.33	0.869	0.723	0.810 (0.721–0.900)	0.764
17-hydroxyprogesterone (17OHP)	0.768 (0.718–0.817)	<0.001	1.04	0.468	1.39	0.757	0.711	0.730 (0.651–0.809)	0.432
11-deoxycortisol (11S)	0.704 (0.647–0.760)	<0.001	0.76	0.407	1.01	0.696	0.711	0.723 (0.642–0.803)	0.707
DOC/P	0.699 (0.645–0.754)	<0.001	0.74	0.383	1.52	0.833	0.550	0.611 (0.521–0.702)	0.102
21-deoxycortisol (21S)	0.683 (0.628–0.738)	<0.001	0.67	0.361	0.14	0.744	0.617	0.654 (0.567–0.740)	0.575
Deoxycorticosterone (DOC)	0.647 (0.687–0.706)	<0.001	0.53	0.494	0.18	0.494	1.000	0.648 (0.558–0.737)	0.988
Cortisone (E)	0.645 (0.587–0.703)	<0.001	0.53	0.317	43.84	0.895	0.422	0.646 (0.558–0.734)	0.981
Progesterone (P)	0.636 (0.578–0.693)	<0.001	0.49	0.257	0.19	0.696	0.561	0.551 (0.460–0.642)	0.123
17OHP/P	0.551 (0.491–0.610)	0.093	0.18	0.140	2.28	0.773	0.367	0.572 (0.481–0.664)	0.699
F/S	0.521 (0.461–0.580)	0.502	0.07	0.106	221.60	0.428	0.678	0.533 (0.442–0.625)	0.818
S/17OHP	0.504 (0.444–0.564)	0.896	0.01	0.108	0.65	0.635	0.472	0.552 (0.461–0.643)	0.390

CI confidence interval, *P value* *P* value for testing the classification performance against a classification at chance level (AUC = 0.5), *K–S statistic* maximum Kolmogorov–Smirnov statistic, *cut-off* cut-off point for classification according to the maximum K–S statistic, AUC_CV_ AUC for the cross-validation sample, *P* *value*_*C*_ *P* value for testing the AUC between the test and verification sample. Steroids and steroids ratios sorted in descending order according to the area under the curve (AUC).

### Sensitivity analysis

In the sample of adolescents with depression, smoking was found to be significantly but only marginally correlated to levels of cortisone (*r*_259_ = 0.11, *P* = 0.03), 17-hydroxyprogesterone (*r*_259_ = 0.14, *P* = 0.006), progesterone (*r*_259_ = 0.14, *P* = 0.009), 21-deoxycortisol (*r*_257_ = 0.18, *P* = 0.002), deoxycorticosterone/progesterone (*r*_259_ = −0.12, *P* = 0.02), and 11-deoxycortisol/17-hydroxyprogesterone (*r*_259_ = −0.10, *P* = 0.04; Supplementary Table 3). Psychotropic medication and 25(OH)D3 levels were significantly but also only marginally correlated with the ratio of corticosterone/deoxycorticosterone (medication: *r*_259_ = −0.12, *P* = 0.02; 25(OH)D3 levels: *r*_259_ = −0.13, *P* = 0.001), and neither steroid was correlated with z-BMI and the SES.

Except for a significant interaction between sample and sex regarding deoxycorticosterone levels (*P* = 0.008; Supplementary Table 4), the same pattern of findings regarding single steroids but also their ratios was confirmed in the restricted psychiatric sample.

When comparing inpatients and daycare patients by a rank-based approach, no difference in cortisol levels between both samples was detected (*F*_1,187_ = 0.57, *P* = 0.45).

## Discussion

Based on a large sample of 261 adolescents with MDD and the reference method of steroid hormone analysis (LC-MS/MS), the present study found altered levels of all studied adrenal steroids related to mineralocorticoid and glucocorticoid synthesis in comparison to subjects from a reference cohort (*N* = 255). Moreover, the corticosterone to deoxycorticosterone ratio reliably discriminated between adolescents from the psychiatric and reference cohort, which especially applied to females. The implications of these findings regarding the pathophysiology of HPA axis dysfunction in MDD and the use of selected adrenal steroids for diagnosing and monitoring MDD will subsequently be outlined.

### ACTH overdrive in adolescent MDD

Several lines of evidence regarding the present study’s results imply a dysfunction of the HPA axis with a chronic ACTH overdrive in adolescent MDD, which will subsequently be outlined concerning glucocorticoid- and mineralocorticoid-related steroids.

First, in line with findings in adults with MDD, mostly relying on post DST results, all glucocorticoid-related C21 steroids, that is, 11-deoxycortisol [6, 7, 32], cortisol [8, 33, 34], and cortisone [6, 35], were increased in adolescent MDD compared to the reference cohort (Figs. 1 and 2). Considering the same pattern of steroid hormone levels in patients with Cushing’s disease [36], these findings imply a chronic ACTH overdrive.

Second, this conclusion is also supported by examining steroid ratios as gross indicators of the activity of those enzymes related to glucocorticoid synthesis. Consistent with what would be expected from the longer-term response to ACTH, the ratios of steroids related to glucocorticoid synthesis (11-deoxycortisol/17-hydroxyprogesterone and cortisol/11-deoxycortisol) were unchanged [37, 38]. However, and in contrast to an unchanged cortisol/11-deoxycortisol ratio in the present study, Holsboer et al. found an increased ratio in a series of small-scale studies including 6 to 23 patients with MDD that all relied on post DST results [33, 34, 39]. The authors suggested that this finding is explained by an ACTH-induced increase in 11β-hydroxylase activity responsible for converting 11-deoxycortisol to cortisol. Even if disregarding methodological differences between the studies by Holsboer et al. and the present study, this hypothesized (patho-)physiology may not hold. As previously discussed by Joyce, Elder [32], who likewise did not detect an increased cortisol/11-deoxycortisol ratio in a study including 37 adults with MDD, the conversion from 11-deoxycortisol to cortisol is not the rate-limiting step in cortisol synthesis. Moreover, considering that steroid hormone levels at each step of glucocorticoid synthesis result not only from their rate of synthesis but also from their further processing and degradation/inactivation, caution is warranted when steroid ratios are solely interpreted as a consequence of enzyme activity. Thus, the overall pattern of findings regarding C21 steroids related to glucocorticoid synthesis and the regulation of corresponding enzymes is interpreted to be in line with chronic ACTH stimulation in adolescent MDD.

Third, 21-deoxycortisol has not yet been studied, to the best of our knowledge, in adult MDD but was found to be decreased in adolescent MDD compared to the reference cohort in the present study. Considering ample support for HPA axis activation in MDD, which in turn results in 21-hydroxylase induction [38, 40], decreased 21-deoxycortisol levels may be an expression of increased 17-hydroxyprogesterone to 11-deoxycortisol conversion due to ACTH stimulation. In line with this, a study in adrenal cells in vitro showed a decrease of about 35% in 21-deoxycortisol levels following ACTH stimulation [41].

Fourth, turning to mineralocorticoids, there were increased corticosterone levels in the present study in adolescent MDD compared to the reference cohort. This is consistent with results from a small-scale study by Seckl, Campbell [42] assessing adrenal steroid hormone levels in seven patients compared to seven controls and most likely implies a chronic ACTH overdrive in MDD when also considering the observation of increased corticosterone levels in patients with Cushing’s disease and the (patho-)physiology of corticosterone synthesis [36]. Corticosterone is not only produced in the zona glomerulosa as a precursor to aldosterone under the control of angiotensin and potassium levels but also in the zona fasciculata along with other glucocorticoids under the control of ACTH [43].

### Deoxycorticosterone—potential pathophysiological implications

Holsboer, Doerr [8] investigated deoxycorticosterone levels in adults with MDD after dexamethasone suppression and found no difference compared to healthy controls. However, the study was limited to 6 female patients and, therefore, underpowered to detect differences in deoxycorticosterone levels between both groups. In contrast, with a sufficiently powered analysis, deoxycorticosterone levels were decreased in adolescent MDD compared to the reference cohort. Interestingly, at the same time, the opposite was found for 11-deoxycortisol. These findings are surprising for two reasons. First, the enzyme 21-hydroxylase (Fig. 1) likely has a higher affinity for progesterone than 17-hydroxyprogesterone [44], and this should favor mineralocorticoid (i.e., deoxycorticosterone) rather than glucocorticoid (i.e., 11-deoxycortisol) synthesis. Second, precursor levels (progesterone) to deoxycorticosterone and 11-deoxycortisol synthesis were increased in MDD compared to the reference cohort making a shortage of substrate supply to the 21-hydroxylase for deoxycorticosterone synthesis improbable. Thus, deoxycorticosterone recruitment to subsequent corticosterone synthesis is likely not the (only) explanation of decreased deoxycorticosterone levels in MDD that may also be related to an aberrant metabolism of neuroactive steroids.

Tetrahydrodeoxycorticosterone (THDOC) is a potent neuroactive steroid with antidepressant effects derived from deoxycorticosterone by peripheral conversion, also in the brain [45]. Interestingly, THDOC serum levels are elevated in adults with MDD compared to controls and are responsive to treatment with fluoxetine [12, 13]. These observations may be related to a central resistance to the antidepressant effects of neuroactive steroids or impaired responsiveness of the HPA axis to negative feedback signals by THDOC, amongst others by decreased corticotropin-releasing hormone mRNA expression in the hypothalamus [15]. Therefore, in either case, it seems feasible that increased THDOC levels in depression arise in expense of deoxycorticosterone due to increased deoxycorticosterone recruitment for THDOC synthesis.

### Corticosterone/deoxycorticosterone—diagnostic implications

In line with a study by Holsboer, Doerr [33] that included 6 depressed patients and based on DST results, there was a cross-validated, increased corticosterone/deoxycorticosterone ratio in adolescent MDD with excellent discriminatory features, especially in females (AUC: 0.957; sensitivity: 0.902; specificity: 0.891). This high diagnostic performance of the corticosterone/deoxycorticosterone ratio points to a significant disorder in the synthesis of adrenal steroids along the pathophysiology outlined above and relates abnormal adrenal functioning to the etiology of MDD, either as cause or consequence. In this regard, results of a recent meta-analysis suggest that only patients with a certain degree of ACTH overdrive seem to benefit from antiglucocorticoid treatment [46], and the corticosterone/deoxycorticosterone ratio may help identify these patients. Moreover, the corticosterone/deoxycorticosterone ratio may qualify to monitor the course of MDD and predict the risk for MDD recurrence in those patients whose underlying pathophysiology has not resolved.

Of note, also corticosterone levels alone proved to be a very good classifier to distinguish between the psychiatric and reference cohort. However, the corticosterone/deoxycorticosterone ratio may combine unique discriminatory features related to each single steroid and, thereby, allow for superior classification performance. While corticosterone levels seem to be especially suitable to distinguish patients with MDD and subjects from the reference cohort, deoxycorticosterone levels may allow for the exclusion of other states of HPA axis dysfunction. As detailed above, Cushing’s syndrome is marked by increased rather than decreased deoxycorticosterone levels observed in MDD. However, whether this also applies to other psychiatric disorders [47] remains to be determined.

### Gender differences in adrenal steroids

The higher classification performance of the corticosterone/deoxycorticosterone ratio was related to a higher corticosterone level and a higher corticosterone/deoxycorticosterone ratio in female psychiatric patients compared to the reference sample, where there were no differences between sexes (Fig. 2).

The same pattern of findings emerged regarding the impact of sex on cortisol levels and the cortisol/cortisone ratio in MDD compared to the reference sample. In healthy controls, there are no differences in corticosterone or cortisol levels between males and females throughout the menstrual cycle [48]. Moreover, to the best of our knowledge, there is no evidence of extra-adrenal and sex-specific corticosterone or cortisol synthesis [37]. Also, a most recent meta-analysis in adults does not support sex-specific responsiveness of the HPA axis to explain this finding [40]. Thus, the underlying pathophysiology of sex-specific changes of steroid hormone levels in MDD has yet to be determined. Given an increased prevalence of MDD in females, it may be worthwhile to gain a better understanding of this observation.

### Limitations

In the psychiatric and reference cohort, adrenal steroids were determined from blood samples obtained in the early morning by a single-point measurement in a clinical setting. However, the anticipation of venipuncture and also hospitalization may activate the HPA axis in a substantial but unpredictable number of subjects which is not accounted for by this approach. Despite these concerns, there was no evidence of an effect of hospitalization on cortisol levels, used as a marker steroid of HPA axis activation, when comparing inpatients and daycare patients [31]. Moreover, according to the central limit theorem, the risk of oversampling individuals with heightened cortisol reactivity, such as in anticipation of venipuncture, reduces with more than 30 observations, which is supported by the distribution of cortisol levels in the present study (Supplementary Fig. 2).

When comparing steroid hormone levels between the reference cohort and adolescents with MDD, we could not account for confounders except for age and sex. This also applied to the depression status of the reference cohort. However, based on a representative study of German children and adolescents, a prevalence of depression not higher than about 6% is expected in the reference cohort, also when considering the age distribution in this sample [49]. Moreover, in adolescents with MDD, smoking status and the intake of psychotropic medication evidenced only marginal correlations with steroid hormone levels, and all findings regarding steroid hormone levels were confirmed in the restricted psychiatric sample, excluding those who reported smoking and psychotropic medication on admission.

While plasma samples were analyzed in the reference cohort, serum samples were subjected to analysis in the psychiatric sample. However, using different blood matrices should not have significantly affected LC-MS/MS results. As reviewed by Ceglarek et al. [23], plasma and serum samples were found to only marginally differ regarding steroid levels, especially when analyzed by LC-MS/MS.

Also, neuroactive steroids other than THDOC may be related to adolescent MDD. Unfortunately, the present LC-MS/MS profile did not allow for a direct measurement of some well-established neuroactive steroids implicated in depression, especially allopregnanolone and its 3β-epimer isopregnanolone [50]. This is a limitation, since brexanolone, an intravenous formulation of allopregnanolone, was approved in 2019 to treat postpartum depression, and zuranolone, a formulation of allopregnanolone with high oral bioavailability for once-daily use, is in phase III clinical trials.

## Conclusions and future perspectives

Results of the present study suggest a chronic ACTH overdrive in adolescent MDD. Moreover, decreased deoxycorticosterone levels in this study could either indicate an impaired HPA axis feedback loop or a central resistance to the antidepressant effects of THDOC. In addition, the corticosterone/deoxycorticosterone ratio was found to provide excellent diagnostic features to discriminate between adolescents with MDD and subjects of the reference cohort, which especially applied to females. This finding highlights a disorder of adrenal steroid synthesis in adolescent MDD, likely related to its etiology. Along this line of reasoning, the corticosterone/deoxycorticosterone ratio may help identify patients who may benefit from antiglucocorticoid treatment and also those at risk for recurrence when the underlying pathophysiology has not resolved.

However, considering that this is the first, well-powered cross-sectional study to address multiple adrenal steroids in adolescent MDD, there is a need for replication and a longitudinal study to relate the detected steroid profile abnormalities to adolescent MDD causally.

Future studies should determine deoxycorticosterone and THDOC to provide a direct and better understanding of their relationship and to disentangle possible explanations of increased THDOC levels in MDD.

Considering the potential clinical implications of altered neuroactive steroid levels in MDD as above-mentioned, subsequent studies in adolescents with MDD should directly address neuroactive steroids, also to assess the rationale for using these drugs, especially allopregnanolone-derivatives, in these patients.

In view of the distributional properties of steroid hormones and high intercorrelations among steroids and their ratios in the present study, a univariate approach robust to outliers and non-normality was chosen. Future studies might consider analyzing data by more robust multivariable approaches, including machine-learning algorithms, that are under active development [51] and might use present findings as a rationale for model building and preselection among highly correlated steroids and steroids ratios.

## Supplementary information

Supplementary Material
