# Supplementary material for: The adrenal steroid profile in adolescent depression: a valuable bio-readout?
Source: Transl Psychiatry. 2022 Jun 18;12:255. doi: 10.1038/s41398-022-01966-2 (PMC9206671; doi:10.1038/s41398-022-01966-2)
Supplement: Supplementary file 1 — Supplementary Material [file 41398_2022_1966_MOESM1_ESM.docx]

**Supplementary Material**

**The Adrenal Steroid Profile in Adolescent Depression: A Valuable Bio-Readout?**

**Outline**

1. Table S1 – Detailed ROC Results – Restricted Sample
2. Table S2 – Bivariate Correlations – Total Sample
3. Table S3 - Ranked ANOVA Results - Restricted Sample
4. Figure S1 – Full Range Boxplots for B, DOC/P, F/S, and B/DOC
5. Figure S2 - Distribution of Cortisol Levels - Total Sample

| **Table S1** - ROC Results – Restricted Sample | | | | | | | |
| --- | --- | --- | --- | --- | --- | --- | --- |
|  | AUC (95%-CI) | P-value | Cohen's d | K-S statistic | cut-off | sensitivtiy | specificity |
| B_DOC |  |  |  |  |  |  |  |
| total | 0.935 (0.910 - 0.957) | <.001 | 2.14 | 0.732 | 25.16 | 0.874 | 0.858 |
| male | 0.844 (0.773 - 0.915) | <.001 | 1.43 | 0.578 | 20.36 | 0.816 | 0.761 |
| female | 0.965 (0.946 - 0.985) | <.001 | 2.56 | 0.820 | 25.16 | 0.945 | 0.874 |
| corticosterone (B) |  |  |  |  |  |  |  |
| total | 0.915 (0.886 - 0.945) | <.001 | 1.94 | 0.714 | 9.80 | 0.792 | 0.922 |
| male | 0.817 (0.736 - 0.898) | <.001 | 1.28 | 0.592 | 6.71 | 0.816 | 0.776 |
| female | 0.954 (0.930 - 0.979) | <.001 | 2.38 | 0.803 | 11.91 | 0.836 | 0.967 |
| cortisol (F) |  |  |  |  |  |  |  |
| total | 0.841 (0.800 - 0.881) | <.001 | 1.41 | 0.587 | 351.78 | 0.811 | 0.775 |
| male | 0.773 (0.641 - 0.824) | <.001 | 1.06 | 0.439 | 266.82 | 0.857 | 0.582 |
| female | 0.880 (0.837 - 0.923) | <.001 | 1.6 | 0.686 | 353.00 | 0.891 | 0.795 |
| F/E |  |  |  |  |  |  |  |
| total | 0.781 (0.734 - 0.828) | <.001 | 1.10 | 0.507 | 6.33 | 0.755 | 0.752 |
| male | 0.681 (0.583 - 0.779) | <.001 | 0.67 | 0.303 | 4.73 | 0.796 | 0.507 |
| female | 0.823 (0.772 - 0.875) | <.001 | 1.31 | 0.625 | 6.33 | 0.864 | 0.762 |
| 17-hydroxyprogesterone (17OHP) | 0.748 (0.699 - 0.798) | <.001 | 0.95 | 0.423 | 1.39 | 0.748 | 0.674 |
| 11-deoxycortisol (11S) | 0.723 (0.667 - 0.778) | <.001 | 0.84 | 0.407 | 1.03 | 0.673 | 0.734 |
| 21-deoxycortisol (21S) | 0.689 (0.636 - 0.742) | <.001 | 0.70 | 0.377 | 0.14 | 0790 | 0.587 |
| deoxycorticosterone (DOC) | 0.677 (0.607 - 0.728) | <.001 | 0.65 | 0.503 | 0.18 | 0.503 | 1,00 |
| DOC/P | 0.675 (0.620 - 0.730) | <.001 | 0.64 | 0.355 | 1.52 | 0.809 | 0.546 |
| cortison (E) | 0.659 (0.604 - 0.713) | <.001 | 0.58 | 0.328 | 45.48 | 0.855 | 0.472 |
| progesterone (P) | 0.604 (0.546 - 0.662) | <.001 | 0.37 | 0.209 | 0.39 | 0.484 | 0.725 |
| 17OHP/P | 0.558 (0.500 - 0.616) | .05 | 0.21 | 0.145 | 1.86 | 0.824 | 0.321 |
| S/17OHP | 0.552 (0.492 - 0.612) | .09 | 0.19 | 0.165 | 0.82 | 0.541 | 0.624 |
| F/S | 0.527 (0.467 - 0.587) | .38 | 0.10 | 0.118 | 305.68 | 0.554 | 0.564 |
| Steroids and steroids ratios in the *restricted* sample sorted in descending order according to the area under curve (AUC). CI = confidence interval, P-value = p-value for testing the classification performance against a classification at chance level (AUC = 0.5), K-S statistic = maximum Kolmogorov-Smirnov statistic, cut-off = cut-off point for classification according to the maximum K-S statistic. | | | | | | | |

|  | smoking | psychotropic medication | 25(OH)D3 | z-BMI | BDI-II | SES | B | E | 17OHP | F | P | DOC | 11S | 21S | B_DOC | F/S | 17OHP/P | DOC/P | S/17OHP | F/E |
| --- | --- | --- | --- | --- | --- | --- | --- | --- | --- | --- | --- | --- | --- | --- | --- | --- | --- | --- | --- | --- |
| smoking | - |  |  |  |  |  |  |  |  |  |  |  |  |  |  |  |  |  |  |  |
| psychotropic  medication | 0.04 | - |  |  |  |  |  |  |  |  |  |  |  |  |  |  |  |  |  |  |
| 25(OH)D3 | -0.03 | -0.03 | - |  |  |  |  |  |  |  |  |  |  |  |  |  |  |  |  |  |
| z-BMI | 0.03 | 0.00 | -0.07 | - |  |  |  |  |  |  |  |  |  |  |  |  |  |  |  |  |
| BDI-II | 0.11* | 0.06 | -0.05 | 0.07 | - |  |  |  |  |  |  |  |  |  |  |  |  |  |  |  |
| SES | -0.10* | -0.01 | 0.21** | -0.08 | 0.02 | - |  |  |  |  |  |  |  |  |  |  |  |  |  |  |
| B | 0.02 | -0.04 | 0.09* | -0.03 | 0.02 | 0.02 | - |  |  |  |  |  |  |  |  |  |  |  |  |  |
| E | 0.11* | -0.07 | -0.01 | -0.08 | 0.03 | 0.06 | -0.03 | - |  |  |  |  |  |  |  |  |  |  |  |  |
| 17OHP | 0.14** | -0.01 | -0.04 | 0.03 | -0.08 | -0.01 | 0.30** | 0.11** | - |  |  |  |  |  |  |  |  |  |  |  |
| F | 0.00 | -0.08 | 0.12** | -0.01 | 0.04 | 0.05 | 0.56** | 0.12** | 0.22** | - |  |  |  |  |  |  |  |  |  |  |
| P | 0.14** | -0.01 | -0.03 | 0.03 | 0.06 | 0.04 | 0.23** | 0.10* | 0.25** | 0.15** | - |  |  |  |  |  |  |  |  |  |
| DOC | -0.01 | 0.08 | -0.04 | -0.05 | -0.08 | -0.05 | 0.34** | 0.07 | 0.30** | 0.20** | 0.18** | - |  |  |  |  |  |  |  |  |
| 11S | 0.04 | 0.01 | 0.02 | 0.00 | -0.04 | 0.02 | 0.39** | 0.09* | 0.29** | 0.24** | 0.21** | 0.33** | - |  |  |  |  |  |  |  |
| 21S | 0.18** | -0.02 | 0.08 | 0.00 | 0.04 | 0.01 | 0.03 | 0.04 | 0.04 | 0.01 | 0.06 | -0.04 | 0.18** | - |  |  |  |  |  |  |
| B/DOC | 0.01 | -0.12* | 0.13** | 0.01 | 0.06 | 0.04 | 0.57** | -0.05 | 0.11* | 0.46** | 0.10* | -0.13** | 0.18** | 0.04 | - |  |  |  |  |  |
| F/S | -0.04 | -0.04 | 0.02 | -0.01 | 0.03 | 0.01 | -0.16** | -0.02 | -0.19** | 0.04 | -0.15** | -0.24** | -0.73** | -0.20** | 0.01 | - |  |  |  |  |
| 17OHP/P | -0.02 | -0.01 | -0.01 | 0.02 | -0.07 | -0.05 | -0.06 | -0.03 | 0.15** | -0.04 | -0.62** | 0.01 | -0.02 | -0.02 | -0.03 | 0.01 | - |  |  |  |
| DOC/P | -0.12* | 0.06 | 0.01 | -0.05 | -0.09* | -0.06 | -0.05 | -0.09* | -0.10* | -0.05 | -0.68** | 0.18** | -0.04 | -0.06 | -0.18** | 0.01 | 0.59** | - |  |  |
| S/17OHP | -0.10* | 0.02 | 0.04 | -0.03 | 0.00 | 0.04 | 0.15** | -0.01 | -0.22** | 0.07 | 0.00 | 0.07 | 0.49** | 0.14** | 0.10* | -0.50** | -0.14** | 0.04 | - |  |
| F/E | -0.08 | -0.01 | 0.11** | 0.04 | 0.01 | 0.02 | 0.53** | -0.27** | 0.14** | 0.61** | 0.09* | 0.15** | 0.17** | -0.02 | 0.47** | 0.05 | -0.02 | -0.01 | 0.08 | - |

**Table S2**. Robust Correlations between Confounders and Steroids

Kendall’s τ_b_ for the variables outlined. * <.05, ** <.01. SES = socioeconomic status, B = corticosterone, E = cortisone, 17OHP = 17-hydroxyprogesterone, F = cortisol, P = progesterone, DOC = deoxycorticosterone, 11S = 11-deoxycortisol, 21S = 21-deoxycortisol.

| **Table S3.** Ranked ANOVA Results **-** Restricted Sample | | |  |  |  |  |
| --- | --- | --- | --- | --- | --- | --- |
|  |  | sample |  | sex |  | sample x sex |
| steroid | F | P | F | P | F | P |
| progesterone (P) | 9.53 | .002 | 10.74 | .001 | 0.29 | .59 |
| 17-hydroxyprogesterone (17OHP) | 61.36 | <.001 | 42.78 | <.001 | 0.41 | .52 |
| desoxycorticosterone (DOC) | 33.34 | <.001 | 14.50 | <.001 | 7.15 | .008 |
| corticosterone (B) | 339.69 | <.001 | 45.41 | <.001 | 66.58 | <.001 |
| 21-desoxycortisol (21S) | 13.78 | <.001 | 3.36 | .07 | 3.37 | .07 |
| 11-desoxycortisol (11S) | 69.56 | <.001 | 0.26 | .61 | 3.52 | .06 |
| cortisol (F) | 148.23 | <.001 | 10.15 | .002 | 23.92 | <.001 |
| cortisone (E) | 19.94 | <.001 | 2.45 | .12 | 2.30 | .13 |
| 17OHP/P | 0.09 | .77 | 86.98 | <.001 | 3.37 | .07 |
| DOC/P | 35.63 | <.001 | 40.64 | <.001 | 3.32 | .07 |
| B/DOC | 468.44 | <.001 | 100.89 | <.001 | 119.43 | <.001 |
| 11S/17OHP | 2.68 | .10 | 14.71 | .005 | 0.29 | .59 |
| F/11S | 0.96 | .33 | 1.25 | .26 | 0.17 | .68 |
| F/E | 85.18 | <.001 | 16.39 | <.001 | 14.91 | <.001 |
| Results for the comparison of means between the restricted psychiatric sample and controls (IV: sample); sample x sex = interaction between both IVs, P= P-value. Between group degrees of freedom (df) = 1, within-group df = 373 for all analysis except 21S (df_within_ = 371). | | | | | | |


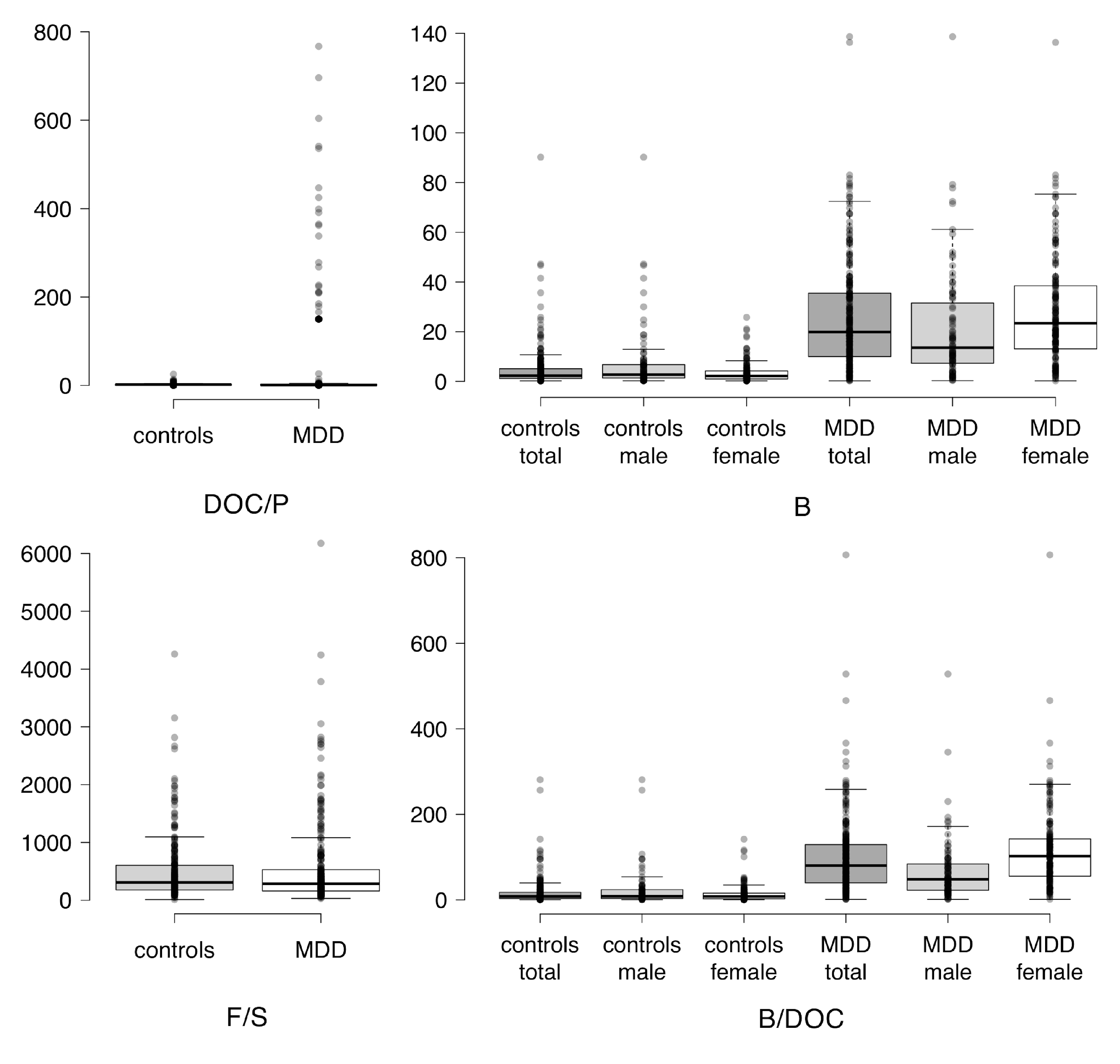


**Figure S1.** Boxplots showing the full range of values for corticosterone and the ratios of deoxycorticosterone/progesterone, cortisol/11-deoxycortisol, and corticosterone/deoxycorticosterone, separately for controls and adolescents with MDD and sex, if applicable (x-axis). The y-axis corresponds to nmol/l concerning corticosterone and is unit-free concerning steroid ratios.


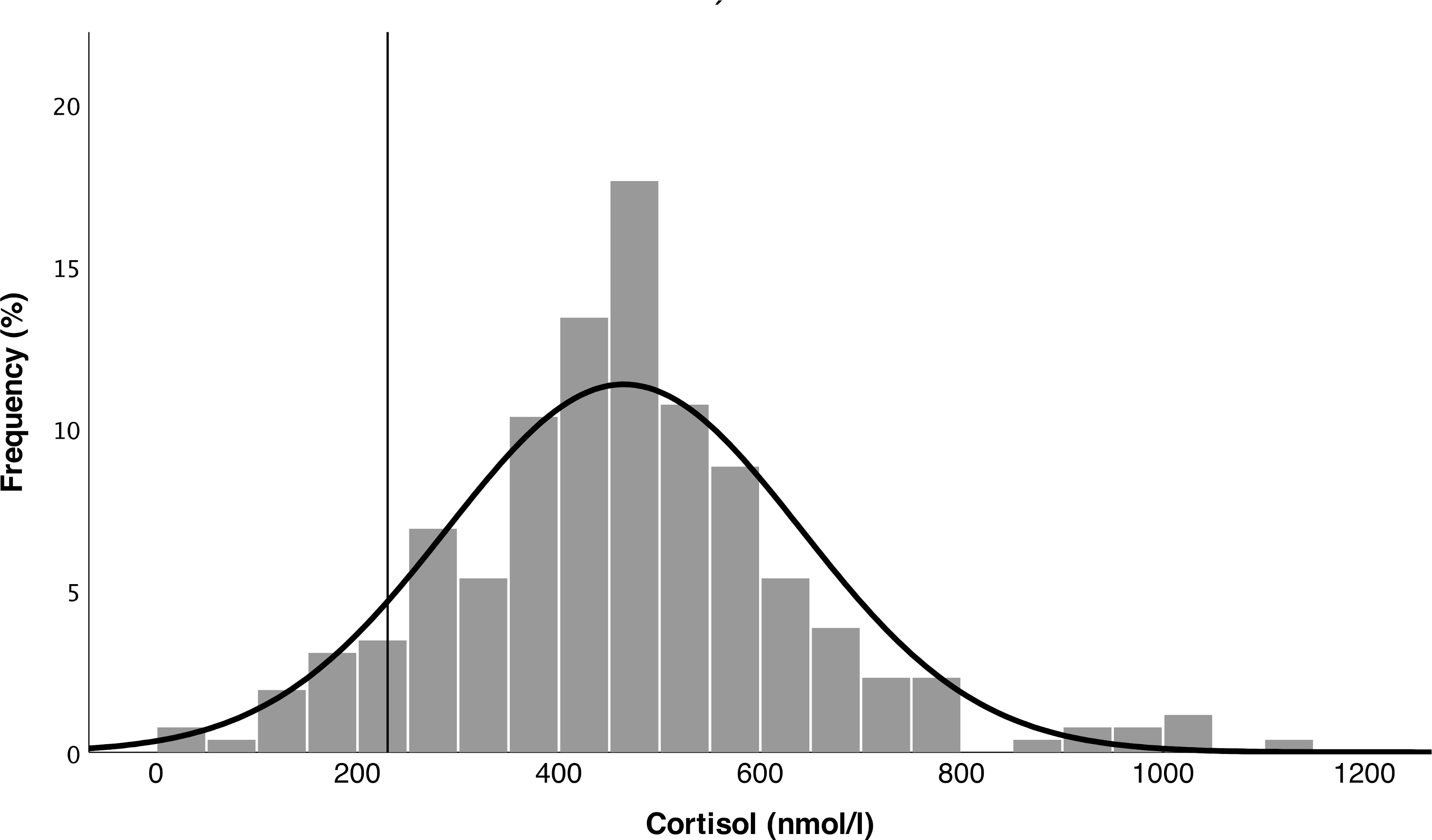


**Figure S2.** Distribution of cortisol levels in patients with MDD with an overlaid normal distribution curve. The vertical line indicates the robust mean according to Huber in controls.
